# Comprehensive Analysis of Expression, Clinicopathological Association and Potential Prognostic Significance of RABs in Pancreatic Cancer

**DOI:** 10.3390/ijms21155580

**Published:** 2020-08-04

**Authors:** Shashi Anand, Mohammad Aslam Khan, Moh’d Khushman, Santanu Dasgupta, Seema Singh, Ajay Pratap Singh

**Affiliations:** 1 Department of Pathology, College of Medicine, University of South Alabama, Mobile, AL 36617, USA; sanand@health.southalabama.edu (S.A.); makhan@health.southalabama.edu (M.A.K.); dasgupta@southalabama.edu (S.D.); seemasingh@health.southalabama.edu (S.S.); 2 Cancer Biology Program, Mitchell Cancer Institute, University of South Alabama, Mobile, AL 36604, USA; 3 Department of Medical Oncology, Mitchell Cancer Institute, University of South Alabama, Mobile, AL 36604, USA; mmkhushman@health.southalabama.edu; 4 Department of Biochemistry and Molecular Biology, College of Medicine, University of South Alabama, Mobile, AL 36688, USA

**Keywords:** *RAB* family genes, pancreatic cancer, UALCAN, clinicopathological, The Human Protein Atlas

## Abstract

RAB proteins (RABs) represent the largest subfamily of Ras-like small GTPases that regulate a wide variety of endosomal membrane transport pathways. Their aberrant expression has been demonstrated in various malignancies and implicated in pathogenesis. Using The Cancer Genome Atlas (TCGA) database, we analyzed the differential expression and clinicopathological association of *RAB* genes in pancreatic ductal adenocarcinoma (PDAC). Of the 62 *RAB* genes analyzed, five *(RAB3A, RAB26, RAB25, RAB21,* and *RAB22A*) exhibited statistically significant upregulation, while five (*RAB6B, RAB8B, RABL2A, RABL2B,* and *RAB32*) were downregulated in PDAC as compared to the normal pancreas. Racially disparate expression was also reported for *RAB3A, RAB25,* and *RAB26*. However, no clear trend of altered expression was observed with increasing stage and grade, age, and gender of the patients. PDAC from occasional drinkers had significantly higher expression of *RAB21* compared to daily or weekly drinkers, whereas *RAB25* expression was significantly higher in social drinkers, compared to occasional ones. The expression of *RABL2A* was significantly reduced in PDAC from diabetic patients, whereas *RAB26* was significantly lower in pancreatitis patients. More importantly, a significant association of high expression of *RAB21, RAB22A,* and *RAB25*, and low expression of *RAB6B, RABL2A,* and *RABL2B* was observed with poorer survival of PC patients. Together, our study suggests potential diagnostic and prognostic significance of RABs in PDAC, warranting further investigations to define their functional and mechanistic significance.

## 1. Introduction

Pancreatic cancer (PC) is the seventh leading cause of cancer mortalities globally, and the third leading cause in the United States [1,2]. According to an estimate made by the American Cancer Society, 57,600 new PC cases will be diagnosed and nearly 47,050 deaths will occur in 2020 in the United States. Indeed, PC is predicted to become the second leading cause of cancer-related death in the United States by 2030 or earlier [3]. 

Pancreatic ductal adenocarcinoma (PDAC) is the most common and lethal subtype of PC, accounting for over 90% of total diagnoses. Most PDACs are diagnosed at late stages (III or IV) due to the lack of recognizable clinical symptoms, leaving limited therapeutic options for treatment [4]. Further, PDAC is a highly aggressive disease that does not respond well to existing therapies due to inherent or acquired resistance, resulting in poor clinical outcomes [5,6,7]. Several genetic aberrations, including *KRAS* mutation, *P53* mutation or deletion, and *SAMD4/DPC4* deletion among several others, have been identified and shown to drive malignant progression [8,9]. However, we have not succeeded in translating this information into effective clinical management approaches. Therefore, we must continue to search for novel molecular targets that could improve early detection, predict the patient’s prognosis, and facilitate the development of mechanism-based therapies.

RAB proteins (RABs) belong to the “RAS superfamily” of small G proteins and comprise the largest subfamily of small GTPases [10,11,12]. A total of 70 RAB proteins have been identified thus far in humans that play indispensable roles in the regulation of vesicle trafficking, including vesicle formation, transport, and the docking and fusion of transport vesicles [12,13,14,15,16]. In particular, RABs regulate the specificity and directionality of membrane-bound cargo traffic to ensure that cargo is delivered to its correct destination [17]. Significant structural homology exists between RAB proteins; however, they have distinct cellular functions and localization [13]. The functions of RABs are controlled by the recruitment of effector or regulatory proteins specific to individual RABs that facilitate the exchange of guanosine diphosphate (GDP) for guanosine triphosphate (GTP) [18]. Some RABs are ubiquitously present, while some have cell type- or compartment-specific functions [19]. Aberrant expression or alterations in the *RAB* genes has been reported in several diseases, including cancer [19,20,21,22,23]. The deregulation of RABs results in the disruption of the regulatory network of vesicle trafficking, which affects cellular growth and behavior and thus facilitates the malignant progression of cancer cells.

The present study examined the expression and clinicopathological association of *RAB* genes in PDAC. Genetic alterations and protein–protein interaction networks of selected upregulated or downregulated *RABs* were also studied. We identified several differentially expressed *RABs* in PDAC, some of which also showed association with the patient’s race as well as diabetes and pancreatitis diagnoses. Furthermore, a significant association of *RAB* genes with patient survival was also found. Finally, we identified low-frequency genetic mutations, amplifications, and deep deletions of *RABs* and their interacting protein networks that suggest their various pathobiological functions in PDAC progression.

## 2. Results

### 2.1. Differential Expression of RAB Transcripts in Pancreatic Tumor Tissues and Association with Clinical Progression

**Figure 1:**
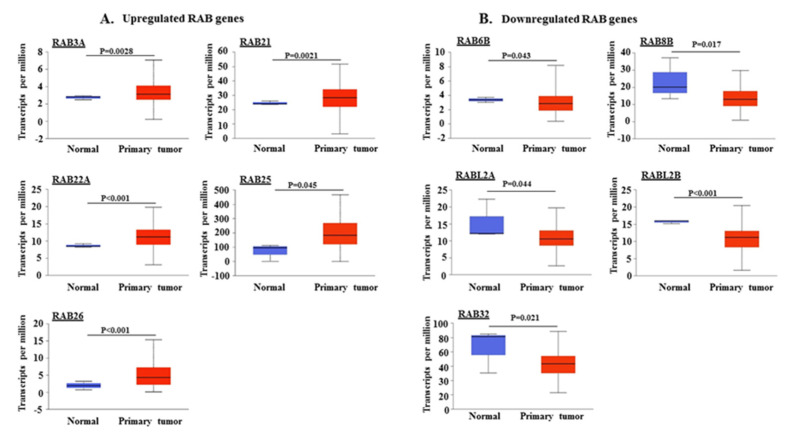
Expression analysis of different *RAB* genes in pancreatic adenocarcinoma patient samples. Expression of different *RAB* genes was analyzed in normal pancreas and primary pancreatic ductal adenocarcinoma (PDAC) samples by using The Cancer Genome Atlas (TCGA) gene expression database on an interactive web portal, UALCAN. Based on the significant (*p* < 0.05) alteration of *RAB* genes in PDAC tissues (*n* = 178) relative to normal tissues (*n* = 4), the *RAB* genes were divided into two subsets: (**A**) upregulated *RAB* genes (*RAB3A*, *RAB21*, *RAB22A*, *RAB25*, and *RAB26*) and (**B**) downregulated RAB genes (*RAB6B*, *RAB8B*, *RABL2A*, *RABL2B*, and *RAB32*).

We analyzed the mRNA expression profiles of 62 *RAB* genes in PDAC using The Cancer Genome Atlas (TCGA) dataset through the UALCAN web portal (http://ualcan.path.uab.edu). A total of 10 RABs exhibited differential expression in primary tumor tissues as compared to the normal pancreas. Five RABs were significantly upregulated, including *RAB3A* (*p* = 0.0028), *RAB21* (*p* = 0.0021), *RAB22A* (*p* < 0.01), *RAB25* (*p* = 0.045), and *RAB26* (*p* < 0.001) (Figure 1A), while the expression of the other five, *RAB6B* (*p* = 0.043), *RAB8B* (*p* = 0.017), *RABL2A* (*p* = 0.044), *RABL2B* (*p* < 0.001), and *RAB32* (*p* = 0.021), was significantly downregulated (Figure 1B). The highest transcripts per million were detected for *RAB25* in both normal and cancerous pancreatic tissues, whereas the lowest transcript levels were observed for *RAB3A*, *RAB6B*, and *RAB26*.

**Figure 2:**
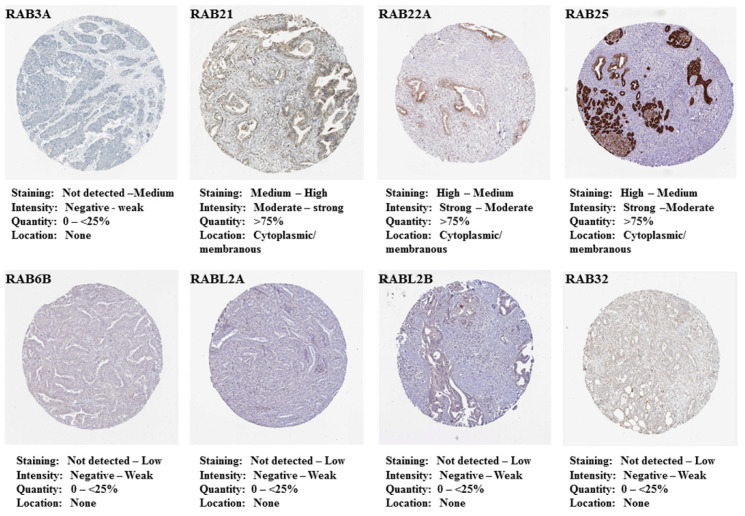
In situ expression of different RABs in pancreatic adenocarcinoma specimens. Expression of upregulated (*RAB3A*, *RAB21*, *RAB22A*, and *RAB25*) and downregulated *RAB* genes (*RAB6B*, *RABL2A*, *RABL2B*, and *RAB32*) was analyzed in PDAC tissues at the protein level using immunohistochemistry data available in The Human Protein Atlas.

We further examined the in situ expression of these *RAB* genes at the protein level using immunohistochemistry data available in The Human Protein Atlas database (https://www.proteinatlas.org/). Correlating with the higher expression at the transcript level, *RAB25* was found to have strong immunostaining in PC tissues, while *RAB21* and *RAB22A* have medium to high staining (Figure 2). Negligible to weak staining, however, is reported for *RAB3A*, *RAB6B*, *RABL2A*, *RABL2B*, and *RAB32* in PDAC tissues. No immunohistochemistry data are available for *RAB26* and *RAB8B* in the database.

We also analyzed the correlation of differentially expressed RABs with PDAC stage and histological grade. Although stage- and grade-specific differences in the expression of some RABs were observed, no clear trend of altered *RAB* expression with increasing stage and grade could be established (Figure S1).

### 2.2. Association of RABs Expression with Race, Gender, and Age of Pancreatic Cancer Patients

**Figure 3:**
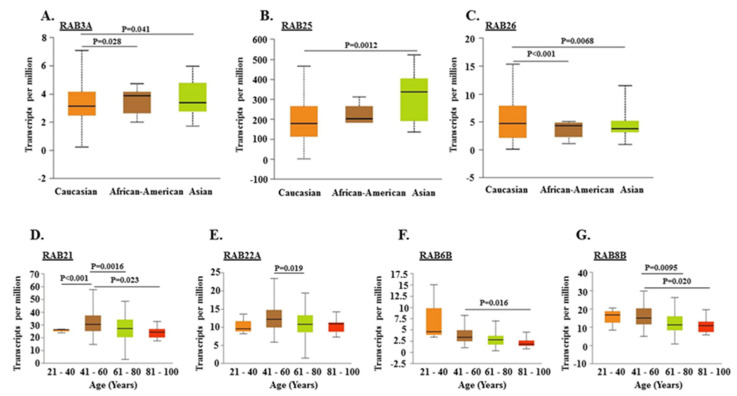
Correlation of differential expression of *RAB* genes with race and age of pancreatic adenocarcinoma patients. Gene expression analysis of *RAB3A*, *RAB25*, and *RAB26* in Caucasian (*n* = 156), African American (*n* = 6), and Asian (*n* = 11) PDAC patients (**A**–**C**). The expression pattern of *RAB21*, *RAB22A*, *RAB6B*, and *RAB8B* was analyzed in different age groups of PDAC patients starting from a younger age range (21–40 years) to older age groups (81–100 years) (**D**–**G**).

Although PDAC appears to afflict both males and females equally, race-specific differences in incidence and mortality are reported [24,25]. Therefore, we examined if the aberrant expression of *RAB* genes in PDAC has any association with patients’ gender, race, and age. We observed racially disparate expression for *RAB3A*, *RAB25*, and *RAB26* in PDAC cases. *RAB3A* and *RAB26* have significantly higher and lower expression, respectively, in PDAC tissues of African American (AA) and Asian racial backgrounds as compared to Caucasian Americans (CA). *RAB25* has significantly elevated expression in PDACs from Asian patients as compared to those from CA patients (Figure 3A–C).

No statistically significant correlation of any of the downregulated *RAB* genes with the patient’s race was observed (data not shown). Similarly, no significant gender-specific association of any of the upregulated or downregulated *RAB* genes was observed (Figure S2). *RAB21* and *RAB22A* showed significantly higher expression in middle-aged patients (41–60 years) compared to younger (21–40 years) and older (61–80 years) patients. The expression of *RAB6B* and *RAB8B* was significantly downregulated in older age groups as compared to the middle-aged patients (Figure 3D–G).

### 2.3. Association of RABs Expression in Pancreatic Tumors with Drinking Habits and Diagnoses of Diabetes and Pancreatitis in Patients

**Figure 4:**
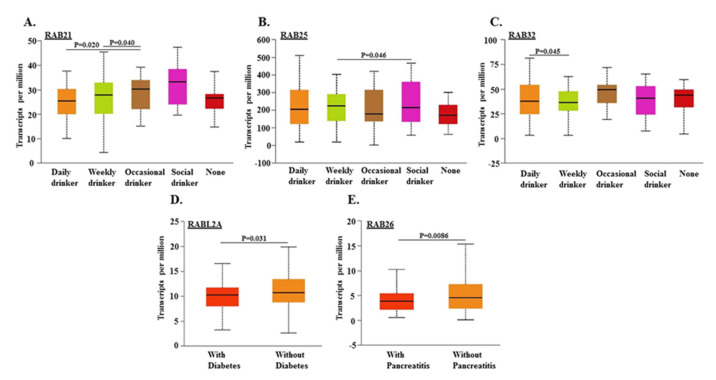
Relationship between differential expression of *RAB* genes in pancreatic tumors with patient’s drinking habits, and diagnoses of diabetes and pancreatitis. Transcript levels of different *RAB* genes (*RAB21*, *RAB25*, *RAB32*, *RABL2A*, and *RAB26*) were correlated with drinking habit (**A**–**C**), diabetes status (**D**), and chronic pancreatitis (**E**) in PDAC patients.

Lifestyle factors, such as drinking habits, and prior diagnoses of diabetes and pancreatitis have been suggested as risk factors for pancreatic cancer [1,26,27]. Therefore, we investigated if there was any association of aberrant *RAB* expression in PDACs with these behavioral and pathological aspects. PDAC cases from occasional drinkers had significantly higher expression of *RAB21* compared to daily or weekly drinkers; however, its expression was the highest (albeit not significant) in social drinkers (Figure 4A).

*RAB25* expression was significantly higher in weekly drinkers as compared to social drinkers, and the expression was the lowest in occasional and non-drinkers but not significant (Figure 4B). Statistically significant differences in transcript levels of *RAB32* were also found in weekly and daily drinkers, but the differences were not significant with occasional and social drinkers (Figure 4C). The expression of *RABL2A* was significantly reduced in PDACs from diabetic patients (Figure 4B), whereas *RAB26* was significantly downregulated in patients with pancreatitis (Figure 4D,E).

### 2.4. Prognostic Potential of RABs Expression in Pancreatic Ductal Adenocarcinoma

**Figure 5:**
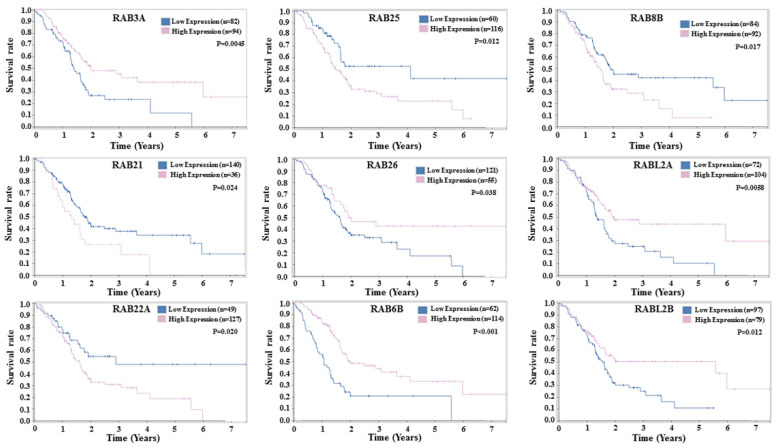
Kaplan–Meier plots for the survival of pancreatic adenocarcinoma patients stratified by the expression level of *RAB* genes. The overall survival curves of the differential expression of upregulated (*RAB3A*, *RAB21*, *RAB22A*, *RAB25*, and *RAB26*) and downregulated (*RAB6B*, *RAB8B*, *RABL2A*, and *RABL2B*) *RAB* genes were analyzed in PDAC patients and correlated with the survival of the patients.

Since dysregulated expression of RABs can impact tumor cell growth and aggressiveness, we investigated its association with patient’s survival. The overall survival (OS) of patients was compared between patients having the high or low expression of various differentially expressed *RAB* genes. Kaplan–Meier plots drawn from the TCGA datasets demonstrated a significant association of several *RAB* genes with the survival of PDAC patients (Figure 5).

Among the 10 differentially expressed RABs, 9 had a significant association with the patient’s survival. High transcript levels of *RAB21*, *RAB8B*, *RAB22A*, and *RAB25* exhibited significant association with a reduced overall survival of PDAC patients. In contrast, high transcript levels of *RABL2A*, *RABL2B*, *RAB6B*, *RAB3A*, and *RAB26* significantly associated with better overall survival of PDAC patients. No significant association of *RAB32* was detected with the patient’s survival (data not shown). It is interesting to note that although *RAB3A* and *RAB26* have an upregulated expression in PC and *RAB8B* has downregulated expression, they unexpectedly exhibit positive and negative associations, respectively, with patient survival.

### 2.5. Genomic Alterations in RAB Genes Associated with Pancreatic Cancer

**Figure 6:**
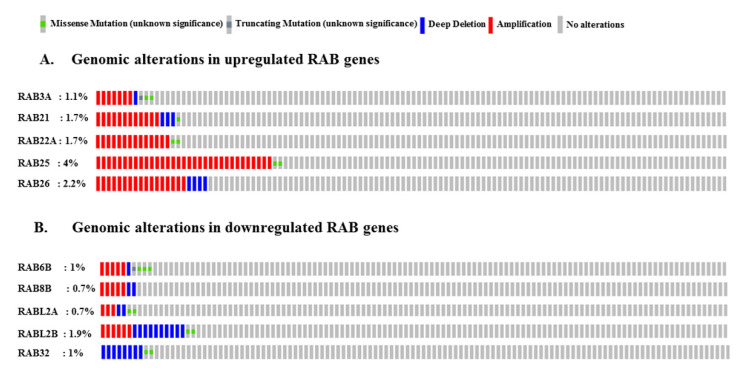
Genomic alterations in differentially expressed *RAB* genes identified in pancreatic adenocarcinoma: OncoPrint analysis of upregulated (**A**) and downregulated (**B**) *RAB* genes using the c-BioPortal revealed extent of gene amplification, deep deletions, and nucleotide substitutions associated with pancreatic cancer.

Altered expression of genes can result from gene amplification or deletion besides aberrant transcriptional regulation. Further, altered gene function can also result from gene mutations. Therefore, we analyzed these genomic alterations in differentially expressed *RAB* genes by using cBioPortal (https://www.cbioportal.org). Among the upregulated *RAB* genes, *RAB25* showed a 4% genetic alteration rate, followed by *RAB26* (2.2%), *RAB21* (1.7%), *RAB22A* (1.7%), and *RAB3A* (1.1%). Most of these alterations were associated with gene amplification; however, deep deletions (*RAB3A*, *RAB21*, and *RAB26*), missense mutations (*RAB3A*, *RAB21*, *RAB22A*, and *RAB25*), and truncating mutation (*RAB3A*) were also reported (Figure 6A).

Interestingly, gene amplification was also reported for some of the downregulated *RAB* genes (*RAB6B*, *RAB8B*, *RABL2A*, and *RABL2B*) (Figure 6). *RABL2B* exhibits the highest rate of deep deletions (1%), followed by *RAB32* (0.85%), *RABL2A* (0.2%), *RAB8B* (0.2%), and *RAB6B* (0.1%). Furthermore, missense mutations (*RAB6B*, *RABL2A*, *RABL2B*, and *RAB32*) and truncating mutations (*RAB6B*) were also reported (Figure 6B).

### 2.6. Protein–Protein Interaction Network of Selected RABs

**Figure 7:**
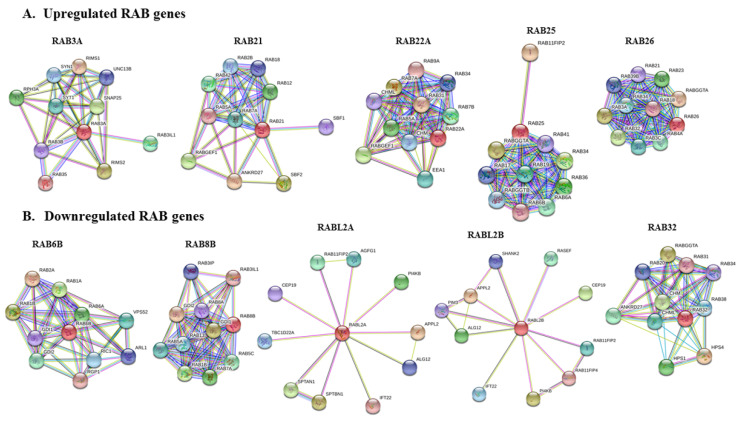
The protein–protein interaction networks of (**A**) upregulated or (**B**) downregulated RAB proteins constructed using STRING.

Protein–protein interaction (PPI) networks were constructed for differentially expressed RABs using STRING (https://string-db.org/) to predict their potential pathobiological significance. Analysis of both known and predicted interactions of upregulated and downregulated RABs revealed several interacting partners, including other RAB GTPase and associated effector and regulatory proteins (Figure 7A,B). A common interaction of Rab GTPase-associated effector proteins involved in the regulation of GDP/GTP reaction exchange (RIMS1, RIMS2, RABGEF1, ANKRD27, SBF1, SBF2, RIC1, RGP1, RAB3IP, RAB3IL1, HPS1, and HPS4) was observed for RAB3A, RAB21, RAB22A, Rab6B, RAB8B, and RAB32.

Similarly, proteins involved in the geranylgeranylation of RAB protein such as CHML, CHM, RABGTA, and RABGTB were also identified as interacting partners for RAB22A, RAB25, RAB26, and RAB32. Of note, some of the downregulated RAB proteins were identified to interact with the same partners that also interact with the upregulated RABs, suggesting complex molecular crosstalk within the RAB functional network. We have summarized the predicted functions of different RAB proteins (Table 1).

## 3. Discussion

Cancer is a progressive genetic disease that begins with the amplification or activating mutations in the oncogenes or deletion or inactivating mutations in the tumor suppressor genes [47,48]. As cancer cells evolve, they accumulate additional genetic aberrations as well as transcriptionally activate or suppress other functionally relevant tumor promoter or suppressor genes that support their malignant progression [49,50]. RAB proteins are an indispensable component of the membrane trafficking system that controls secretion, transport, recycling, and degradation of many tumor-associated proteins such as beta-integrins, epidermal growth factor receptor (EGFR), and matrix metalloproteinases (MMPs), among several others [51,52,53,54]. Further, the role of RABs in the shedding of extracellular vesicles has also been reported [55,56,57,58]. Aberrant expression of RABs is reported in various cancers, and they are shown to act either as a tumor promoter or suppressor in a context-dependent manner [59,60,61,62,63].

Among the upregulated RAB genes identified in PDAC, a higher expression of *RAB3A* is also reported in glioblastoma multiforme, where it promotes cell proliferation [64]. Moreover, its effector protein RAB3IP is involved in insulin exocytosis from pancreatic β cells [65] and promotes cell proliferation via inhibiting autophagy in gastric cancer [66]. RAB21 is known to regulate adhesion molecules and endosomal traffic of β-integrins [67] and promote the proliferation of glioblastoma [68] and breast cancer cells [69]. Further, RAB21 mediates acute pancreatitis through interaction with the TRAF3-MKK3 complex [70]. RAB22A is reported to promote the biogenesis of recycling endosomes [71] and regulate the sorting of membrane proteins such as transferrin to recycling endosomes [32]. RAB22A acts as a prognostic marker in breast cancer [72] and inhibits invasion and metastasis of breast cancer cells through secretion of exosomes [73]. It is also shown to be involved in the pathogenesis of colon cancer [74], renal cell carcinoma [75], and osteosarcoma [76]. RAB25 is involved in the recycling of proteins from late endosomes to the plasma membrane during cell migration [77]. The tumorigenic role of RAB25 is reported in non-small cell lung cancer [78], gastric cancer [79], and ovarian cancer [80], whereas some report its tumor suppressor function in breast [81] and colon cancers [82]. RAB26 is generally found in secretory cells such as pancreatic acinar cells and controls lysosomal traffic and mitochondrial localization and trafficking of synaptic vesicles [36,37].

Cancer-related information is scarce for the *RAB* genes that we found to be downregulated in PDAC; however, these RABs are reported to play essential roles in cellular physiology. RAB6B is expressed in a cell-type-specific manner and is involved in retrograde (Golgi to Endoplasmic reticulum) trafficking of proteins [83]. RAB8B is essential for the apical transport pathways and helps in intracellular vesicle docking and fusion to their target membrane [40,84]. Both RABL2A and RABL2B are known for intra-flagellar ciliary transport and control ciliary G-protein-coupled receptor (GPCR) trafficking [43,44]. Further, RAB32 is reported to be expressed in secretory cells, regulate autophagy and mitochondrial dynamics, and reside on lysosomes [45,85,86].

Racial disparities in cancer incidence and clinical outcomes are reported for many cancers, including PC [87]. While cancer health disparities could result from multiple factors, molecular and biological differences have been suggested to be involved as well [88]. Along those lines, we observed racially disparate expression of three RAB genes (*RAB3A*, *RAB25*, and *RAB26*). Therefore, it will be interesting to further investigate their functional significance in PC using appropriate model systems. Age, alcohol consumption, and prior diagnoses of diabetes and pancreatitis are suggested to be risk factors for developing PC [1]. Most PCs are diagnosed in patients over 60 years old, and they rarely develop in younger adults (less than 40 years old) [2,89]. In our study, we found significant differences in levels of some upregulated (*RAB21* and *RAB22A*) and downregulated (*RAB6B* and *RAB8B*) RABs between age groups, but the pattern is not distinct enough to predict an etiological association. High alcohol consumption is also suggested as a risk factor for PC development [90]. We, however, did not observe a clear-cut suggestive association of RAB dysregulation with drinking habits, although statistically significant differences in levels were observed between daily, weekly, occasional, and social drinkers for some RABs. Type I and II diabetes and pancreatitis status are considered as potential risk factors of PC as well [1]. We found that *RABL2A* had significantly lower expression in PC patients with diabetes. It would be interesting to investigate its functional significance. We also found a significantly lower expression of *RAB26* in PC patients with pancreatitis, which could suggest its regulation by inflammatory mediators.

We found a significant association of differentially expressed RABs with the survival of PDAC patients. Interestingly, some of the RABs (*RAB3A*, *RAB26*, and *RAB8B*) displayed an unexpected association with patients’ survival. Despite being overexpressed in PC, high *RAB3A* and *RAB26* have a positive association with patient survival, while low levels of the downregulated *RAB8B* have a negative association with PC patient survival. This could be due to the altered functions of these RABs due to genetic alterations or their secondary regulation affecting their protein levels. Supporting this notion, we observed a lack of *RAB3A* immunostaining in PC tissues, suggesting its post-transcriptional regulation by yet unknown mechanisms [91,92]. It is also likely that the functional pathobiological impact of these RABs depends on other interacting proteins that may also be aberrantly expressed in PC. Therefore, functional and mechanistic insights regarding the roles of RABs are needed through laboratory research to precisely understand their pathobiological significance in PC. In summary, our study provides initial data to support further investigations on RAB functions and associated mechanisms to explore their utility as therapeutic targets and clinical biomarkers of diagnostic and prognostic significance.

## 4. Materials and Methods

### 4.1. Gene Expression Analysis Using UALCAN

UALCAN (http://ualcan.path.uab.edu/) is a publicly available web portal that uses TCGA level 3 RNAseq and clinical data to provide the differential expression status of the gene of interest in normal and cancerous tissue and its clinicopathological association. To assess the potential role of RAB proteins in the progression of PC, UALCAN was used to examine the relative transcript levels of various *RAB* genes in normal pancreas and pancreatic tissue samples. *RAB* genes with significant *(p* < 0.05) overexpression or downregulation in PDAC tissues were further examined for their clinicopathological significance. The UALCAN server was also used to analyze the association of upregulated or downregulated *RAB* genes with tumor grade and stage, and patient’s race, gender, age, drinking habits, diabetes, and pancreatitis status.

### 4.2. Prognostic Significance and In Situ Expression of Differentially Expressed RAB Genes

The association of differential expression status of *RAB* genes with patient survival was examined using TCGA database information via The Human Protein Atlas (https://www.proteinatlas.org/). Kaplan–Meir survival plots were drawn for prognostic values of *RAB* genes that had significant up- or downregulation in PC at the mRNA level. *RAB* genes with significant (*p* < 0.05) correlation of changes in survival rate with either higher or lower expression of *RAB* genes were chosen. The Human Protein Atlas stores plenty of information about thousands of proteins regarding their expression and distribution in a variety of cancer tissues. Using this database, we analyzed the protein expression and localization status of selected *RAB* genes in the pancreatic adenocarcinoma tissues.

### 4.3. Genomic Alterations Analysis and Protein–Protein Interaction Network of Selected RAB Genes

c-BioPortal (https://www.cbioportal.org/) is a readily accessible platform for the analysis of various cancer genomics datasets such as TCGA, GEO, and ICGC that provides information related to somatic mutation spectrum (gene amplification, deep deletion, missense mutations, etc.) and changes in mRNA and microRNAs [93,94]. We analyzed the genetic alterations in PC of upregulated and downregulated *RAB* genes using c-BioPortal, and results obtained in ‘Oncoprint’ for each *RAB* gene were downloaded and presented. Further, we used the STRING web interface (https://string-db.org/) [95] to examine the interacting partners of potential *RAB* genes through the construction of protein–protein interaction networks.

## Figures and Tables

**Table 1:** Function and localization of upregulated or downregulated *RAB* genes in pancreatic cancer.

*RAB* Genes	Location	Function	References
**Upregulated RAB Genes**			
*RAB3A*	Synaptotagmins and secretory vesicles	Regulation of exocytosis (Ca^+2^ dependent), secretory vesicle docking, fusion and transport of synaptic vesicles	[28,29]
*RAB21*	Early endosomes, trans Golgi network, cytoplasmic vesicles	Endosomal transport of integrins, cell adhesion and migration, endolysosomal transport	[30,31]
*RAB22A*	Early endosomes, phagosomal membrane, and trans Golgi network	Protein trafficking from early endosomes to late endosomes, phagosome maturation, recycling endosomal biogenesis	[32,33]
*RAB25*	Recycling endosomes	Protein trafficking through recycling endosomes to the plasma membrane, integrin transport	[34,35]
*RAB26*	Synaptic and secretory vesicles	Regulated exocytosis of secretory granules and synaptic vesicles	[36,37]
**Downregulated RAB Genes**			
*RAB6B*	Golgi	Retrograde transport from late endosomes via Golgi to ER	[38,39]
*RAB8B*	Golgi, plasma membrane, phagosome membrane	Apical transport pathways, adherens junction dynamics, from trans Golgi network and recycling endosomes to plasma membrane	[40,41,42]
*RABL2A/RABL2B*	Primary cilia, centriole	Intraflagellar transport and ciliary assembly	[43,44]
*RAB32*	Mitochondria, melanosomes, autophagosomes	Mitochondrial dynamics regulation, transport from trans Golgi network to melanosomes, autophagy	[45,46]

## Supplementary Materials

The following are available online at https://www.mdpi.com/1422-0067/21/15/5580/s1, Figure S1: Association of transcript levels of *RAB3A*, *RAB21*, *RABL2A*, *RABL2B* and *RAB32* with tumor grade and stages of pancreatic adenocarcinoma, Figure S2: Gender-based differential expression of RAB genes.

## Abbreviations

AA	African American
CA	Caucasian American
PDAC	Pancreatic ductal adenocarcinoma
TCGA	The Cancer Genome Atlas
