# Supplementary material for: Comprehensive Analysis of Expression, Clinicopathological Association and Potential Prognostic Significance of RABs in Pancreatic Cancer
Source: Int J Mol Sci. 2020 Aug 4;21(15):5580. doi: 10.3390/ijms21155580 (PMC7432855; doi:10.3390/ijms21155580)
Supplement: Supplementary file 1 [file ijms-21-05580-s001.pdf]

Supplementary information

Comprehensive Analysis of Expression, Clinicopathological Association and Potential Prognostic Significance of RABs in Pancreatic Cancer

Shashi Anand [1,2], Mohammad Aslam Khan [1,2], Moh'd Khushman [3], Santanu Dasgupta [1,2], Seema Singh [1,2,4] and Ajay Pratap Singh [1,2,4,*]

1. Department of Pathology, College of Medicine, University of South Alabama, Mobile, AL 36617, USA; sanand@health.southalabama.edu (S.A.); makhan@health.southalabama.edu (M.A.K.); dasgupta@southalabama.edu (S.D.); seemasingh@health.southalabama.edu (S.S.)
2. Cancer Biology Program, Mitchell Cancer Institute, University of South Alabama, Mobile, AL 36604, USA
3. Department of Medical Oncology, Mitchell Cancer Institute, University of South Alabama, Mobile, AL 36604, USA; mmkhushman@health.southalabama.edu
4. Department of Biochemistry and Molecular Biology, College of Medicine, University of South Alabama, Mobile, AL 36688, USA

* Correspondence: asingh@health.southalabama.edu; Tel.: +1-251-445-9843; Fax: +1-251-460-6994

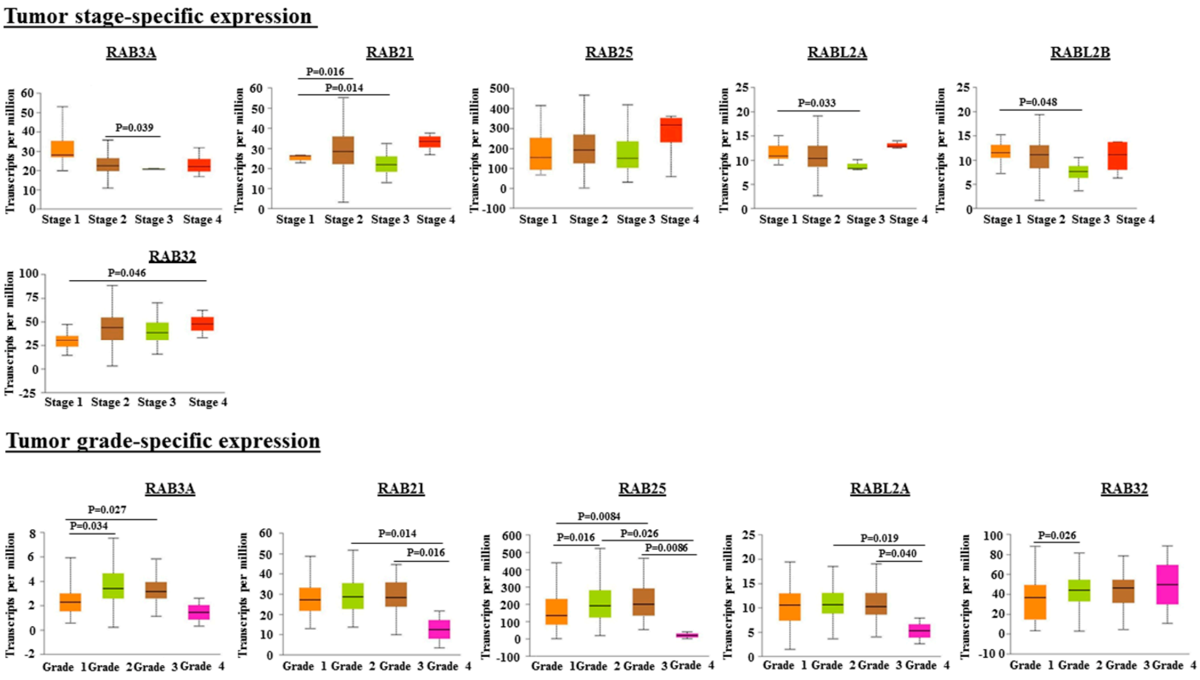

Figure S1. Association of transcript levels of *RAB3A*, *RAB21*, *RAB25*, *RABL2A*, *RABL2B* and *RAB32* with tumor grade and stage of pancreatic adenocarcinoma. Statistically significant tumor stage- and grade-specific association of some *RAB* genes was detected, but no clear pattern of association was evident. Stage 1 (n = 6), stage 2 (n = 146),

stage 3 (n = 4) and stage 4 (n = 4). Grade 1- well differentiated (n = 31), grade 2-moderately differentiated (n = 95), grade 3- poorly differentiated (n = 48) and grade 4- undifferentiated (n = 2).

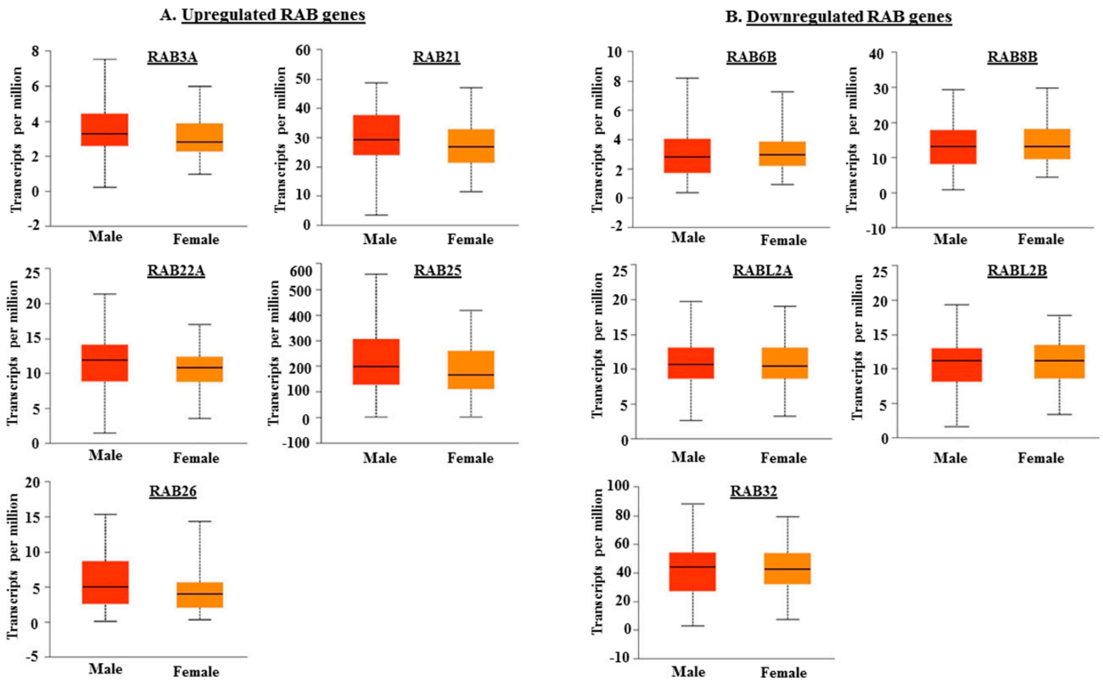

Figure S2. Gender-based differential expression of *RAB* genes. Transcript levels of (**A**) upregulated (*RAB3A, RAB21, RAB22A, RAB25* and *RAB26*) and (**B**) downregulated (*RAB6B, RAB8B, RABL2A, RABL2B* and *RAB32*) RABs in PDAC were examined for gender-specific differences. Male (n = 97); Female (n = 80). No significant association was observed.
